# Assessing Osteopathic Medical Student Knowledge of Common Vitamin and Mineral Deficiencies

**DOI:** 10.7759/cureus.94631

**Published:** 2025-10-15

**Authors:** Autumn A Stevens, Sara-Bethany Weir

**Affiliations:** 1 College of Medicine, Alabama College of Osteopathic Medicine, Dothan, USA; 2 Family Medicine, Alabama College of Osteopathic Medicine, Dothan, USA

**Keywords:** curriculum development, micronutrients, osteopathic, primary care, undergraduate medical education

## Abstract

Many essential micronutrients are commonly deficient in the United States population, resulting in widespread negative health outcomes. Recognition of these deficiencies within primary care settings is paramount, and this training should begin in medical school. Osteopathic medical students are a crucial target for additional nutritional training due to the large number of osteopathic physicians practicing in primary care. Establishing the extent of current knowledge among osteopathic medical students is important for determining the need for additional nutrition education within the medical school curriculum.

This survey-based study utilized a cross-sectional convenience sample of osteopathic medical students at the Alabama College of Osteopathic Medicine (ACOM). Respondents (n = 70) included 34 OMS-I (48.6%), 15 OMS-II (21.4%), 10 OMS-III (14.3%), and 11 OMS-IV (15.7%) students. The study had four objectives: (1) to determine prior exposure to nutritional education and confidence in nutritional knowledge, (2) to evaluate knowledge of the specific benefits of vitamins and minerals, (3) to assess the likelihood of students recommending supplemental nutrition to future patients, and (4) to determine student interest in additional nutritional education. Micronutrients assessed included vitamin B12, vitamin D, magnesium, and folate. All statistical tests utilized a significance value of α = 0.05.

Seventy of 806 students enrolled at ACOM responded to the survey (response rate, 8.7%). No respondents were excluded. Fifty-five (78.6%) students had been exposed to nutrition education, but only 37 (52.9%) students felt somewhat or very confident in their current nutritional knowledge. All p-values were significant when comparing the knowledge of pre-clinical students to post-clinical students. Analysis of variance and Tukey HSD testing revealed significant results when comparing knowledge of specific micronutrient benefits across all students (p < .0001), particularly when comparing knowledge of vitamin D (55, 78.6%) to vitamin B12 (36, 51.4%; p < .01), magnesium (35, 50.0%; p < .01), and folate (33, 47.1%; p < .01). Overall willingness of students to recommend each micronutrient was very high (64 (91.4%), 67 (95.7%), 63 (90.0%), and 65 (92.9%) for vitamin B12, vitamin D, magnesium, and folate, respectively). Sixty-eight (97.1%) surveyed students indicated interest in learning more about micronutrients in their classes.

Many students have had exposure to nutritional coursework, yet few are fully confident in their education. Students demonstrated gains in micronutrient knowledge throughout medical school, but certain micronutrients show superior retention compared with others. Furthermore, students are overwhelmingly interested in recommending these micronutrients to their future patients. Osteopathic students demonstrate a clear need for ongoing education to strengthen recognition of micronutrient deficiencies, with potential for substantial benefits in primary care.

## Introduction

Vitamins and minerals are important micronutrients that are beneficial to an individual’s overall health and well-being. Deficiency of these micronutrients can predispose patients to a variety of diseases and illnesses, with health impacts ranging from mild disruptions to life-threatening complications. Assessing for these deficiencies within the primary care setting and addressing any insufficiencies through dietary adjustments or nutrient supplementation is critical for improving the overall health of our communities and preventing further stress on the healthcare system.

A Western diet is traditionally high in sugar, calories, fat, and sodium, and lacks foods such as fruits and vegetables [1]. Fruits and vegetables are a major source of crucial vitamins and minerals, and a large portion of Americans are prone to deficiencies in these micronutrients due to adherence to a Western diet. Numerous factors perpetuate poor nutrition in America, including a lack of nutritional education and the existence of food deserts, an issue of particular importance in the American South [2,3]. Reduced access to fresh produce and quality meat in food deserts contributes to substantial rates of vitamin and mineral deficiencies in the United States. Poor nutrition plays a significant role in the prevalence of obesity and cardiovascular disease rampant in America, as well as in many additional diseases that lead to poor health outcomes.

The prevalence of vitamin D deficiency among Americans has been found to be as high as 41.6% [4]. Vitamin D is crucial for reducing bone fracture risk, lowering the risk of developing multiple sclerosis, and decreasing overall cancer mortality [5]. Vitamin D can be found in fish, egg yolks, and some mushrooms, as well as absorbed through exposure to sunlight [6]. Vitamin B12 insufficiency may be as high as 12.5% among adults over the age of 19 [7], and is linked to increased rates of anemia, nervous system degeneration, and certain psychiatric illnesses [8]. Vitamin B12 can be found in meat, fish, dairy products, and eggs [9]. Nearly half of all individuals over the age of one in the United States have been found to have insufficient magnesium intake [10]. Magnesium deficiency has been shown to increase the incidence of migraines, Alzheimer’s disease, stroke, hypertension, cardiovascular disease, and type 2 diabetes mellitus [11]. Magnesium can be acquired through consumption of leafy green vegetables, nuts, and whole grains [12]. Due to preventive healthcare efforts to increase folate consumption among women of childbearing age in the 1990s, folate deficiency in the population has decreased to only 0.3% [13]. It remains important to check and maintain folate levels due to its close connection to vitamin B12 deficiency and purine synthesis [14]. Folate can be found in leafy green vegetables, fruits, beans, seafood, eggs, dairy, and meat [15].

The prevalence of insufficiency in these four micronutrients makes them preferred targets for evaluation and correction, but the relative ease of assessing these vitamins and minerals also makes them ideal candidates for further medical education. Vitamin B12, vitamin D, magnesium, and folate levels can all be easily identified in serum, significantly reducing the medical and financial burden of diagnosis [16]. When assessing overall health and well-being in a clinical setting, measuring these four micronutrients provides a sufficient preliminary baseline for the majority of patients.

Recognition of these vitamin inadequacies begins with physician education, with specific emphasis required for students likely to encounter these patients within a primary care setting. Osteopathic medical schools represent a uniquely influential target to bolster micronutrient education, as more than half of all osteopathic medical graduates practice within a primary care setting [17].

Prior research largely focuses on general nutrition knowledge among medical students, such as dietary recommendations and macronutrients, rather than targeting specific micronutrients most likely to benefit a large portion of Americans [18]. Previous literature also addresses student perceptions of the quantity of nutrition education received in school, showing high levels of student interest in additional education [19]. This study has four primary objectives: (1) to determine the level of prior nutritional education and confidence in that education among osteopathic medical students, (2) to assess student knowledge regarding the benefits of four vitamins and minerals (vitamin B12, vitamin D, magnesium, and folate), (3) to gauge the likelihood that these students will recommend micronutrient supplementation to their future patients, and (4) to assess interest in further nutrition education.

Determining the familiarity of students with these specific, commonly deficient micronutrients will help identify the need for further vitamin and mineral education within the medical curriculum and pinpoint specific learning gaps. The latter objectives aim to gauge students’ willingness to learn more about this subject and how they can minimize micronutrient deficiency in their future patients. The researchers hypothesize that osteopathic medical students will not have sufficient knowledge of micronutrients and will require additional education in nutrition, and that students will demonstrate significant willingness to learn about preventing nutritional deficiencies. This article was previously presented as a poster at the 2024 AOA Osteopathic Medical Education Conference (OMED) on September 20, 2024.

## Materials and methods

This was a survey-based study that utilized a cross-sectional convenience sample of first-, second-, third-, and fourth-year osteopathic medical students at the Alabama College of Osteopathic Medicine (ACOM). The study assessed the knowledge of osteopathic medical students on the specific benefits of vitamins and minerals, as well as their likelihood to recommend supplemental nutrition to their future patients. This study was approved by the institutional review board at the Alabama College of Osteopathic Medicine, protocol number 24-05-07-001-ACOM.

Sample

Students enrolled at the Alabama College of Osteopathic Medicine during the 2024-2025 academic year were emailed a survey invitation through ACOM’s Research Department email database. Enrolled students included first, second, third, and fourth year students, with 70 responses in total. The link was emailed to students on July 25, 2024, and was available for students to access through August 24, 2024.

Survey design

Survey questions were developed by both investigators and consisted of four sections: demographics (4 questions), vitamin and mineral knowledge (4 questions), assessment of propensity to prescribe vitamins and minerals (4 questions), and eagerness to learn more information about micronutrients (1 question). The questionnaire is shown in the Appendices. The survey platform Qualtrics was utilized to format the questions and collect responses via an emailed link. Consent to participate was collected in the Qualtrics form prior to viewing the survey questions. The demographic questions assessed each student’s class year, prior education in nutrition, and confidence in their nutrition knowledge. The knowledge-based questions assessed students’ ability to identify the specific functions and benefits of four vitamins and minerals. The likelihood of prescribing these four vitamins and minerals was collected using a four-point rating scale.

Vitamin and mineral selection

Four vitamins and minerals were considered for assessment based on the large degree of deficiency of these micronutrients within the general population. Deficiency in these micronutrients is easily testable through serum tests and can be rectified through oral supplements, easing the barrier for evaluation and administration for the prescriber and patient. The selected micronutrients evaluated were vitamin B12, vitamin D, magnesium, and folate.

Analysis

Organization and analysis of data were performed by the sub-investigator and reviewed by the primary investigator. Data were exported from the Qualtrics platform (Qualtrics, LLC, Provo, Utah) into Microsoft Excel (Microsoft Corporation, Redmond, Washington). Statistical analysis was performed using VassarStats t-test and ANOVA statistical compilation platforms. Power assessment was conducted using Statistics Kingdom (Melbourne, Australia). Graphical data representation and calculation of standard error were completed using Microsoft Excel. A t-test was conducted comparing the percentage of correctly answered vitamin and mineral benefit questions between pre-clinical (OMS-I and OMS-II) and post-clinical students (OMS-III and OMS-IV). An ANOVA test was performed comparing the correctly answered micronutrient benefit questions among all students, assessing for any differences in knowledge between specific vitamins or minerals. All tests utilized a significance value of α = 0.05. Effect sizes for the t-tests were calculated using Cohen’s d formula. Cohen’s criteria for small, medium, and large effect sizes are 0.2, 0.5, and 0.8 or greater, respectively. Effect size for the ANOVA was calculated using eta-squared, with effect size criteria defined as less than 0.01 (small), between 0.01 and 0.06 (medium), and greater than 0.06 (large).

## Results

Participants

The survey was completed by 70 students enrolled at the Alabama College of Osteopathic Medicine. Respondents identified themselves as OMS-I (34, 48.6%), OMS-II (15, 21.4%), OMS-III (10, 14.3%), or OMS-IV (11, 15.7%).

Self-rated nutritional education, confidence, and importance of micronutrients

Responses to questions regarding nutrition education level, confidence in applying that education, and the perceived importance of micronutrients are listed in Table 1. Results show that most students have had some prior exposure to nutrition education (55, 78.6%) through personal research, lectures, or classes in nutrition, but most students were only somewhat confident (35, 50.0%) or somewhat unconfident (24, 34.3%) in the education they received. Students largely viewed micronutrients as important for overall health, with 63 (90.0%) identifying them as somewhat or very important. 

**Table 1:** Nutritional education, confidence level, and importance of micronutrients in osteopathic medical students.

Variables	n (%)
Education level in nutrition	
No nutrition education	15 (21.4%)
Personal research on nutrition	24 (34.3%)
Attended a nutrition lecture	9 (12.9%)
Taken a nutrition class	21 (30.0%)
Formal nutrition degree	1 (1.4%)
Confidence in nutrition education	
Very unconfident	9 (12.9%)
Somewhat unconfident	24 (34.3%)
Somewhat confident	35 (50.0%)
Very confident	2 (2.9%)
Importance of nutrition education	
Very unimportant	5 (7.1%)
Somewhat unimportant	2 (2.9%)
Somewhat important	23 (32.9%)
Very important	40 (57.1%)

Micronutrient knowledge of pre-clinical vs. post-clinical students

**Figure 1:**
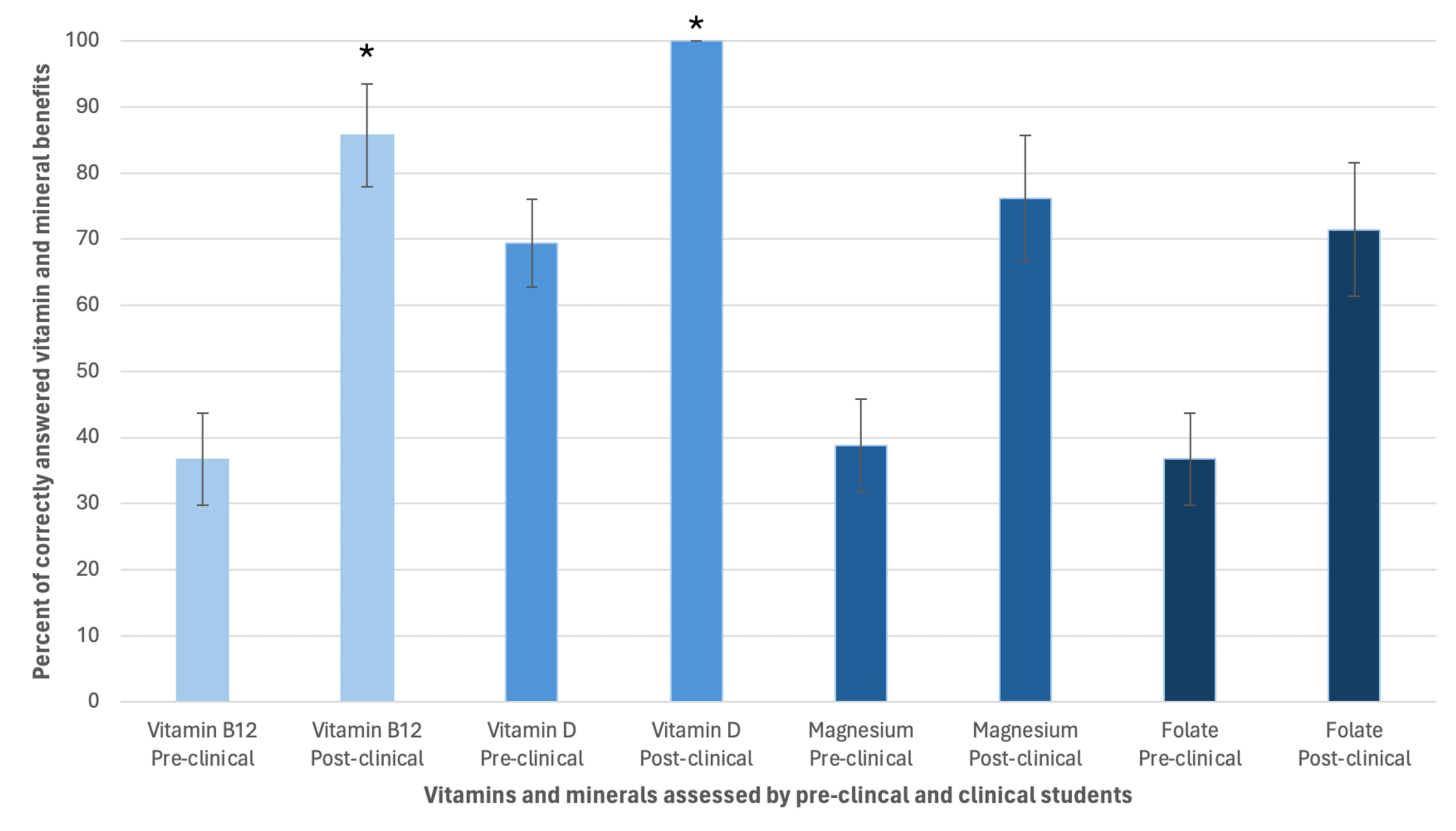
Micronutrient knowledge of pre-clinical and post-clinical osteopathic students. Statistical significance was found for each micronutrient when comparing the knowledge of pre-clinical to post-clinical students, indicating a difference in knowledge between the two groups.

Four two-tailed t-tests were performed comparing the number of pre-clinical students (OMS-I and OMS-II, n = 49) to post-clinical students (OMS-III and OMS-IV, n = 21) who correctly answered questions regarding the specific benefits of vitamin B12, vitamin D, magnesium, and folate. The p-values for each t-test are listed in Table 2. All p-values were statistically significant, indicating a difference in knowledge levels between pre-clinical and post-clinical osteopathic medical students. The results of the four t-tests, along with their respective effect sizes, are displayed in Figure 1. 

**Table 2:** P-values for t-tests comparing micronutrient knowledge of pre-clinical and post-clinical students. All p-values were found to be significant, indicating there is a difference in the knowledge of pre-clinical and post-clinical students for all tested micronutrients.

Vitamin or Mineral Compared	p-value	Effect Size
Vitamin B12	<0.0001	1.01
Vitamin D	0.004	0.66
Magnesium	0.004	0.76
Folate	0.007	0.71

Specific micronutrient knowledge of all students

**Figure 2:**
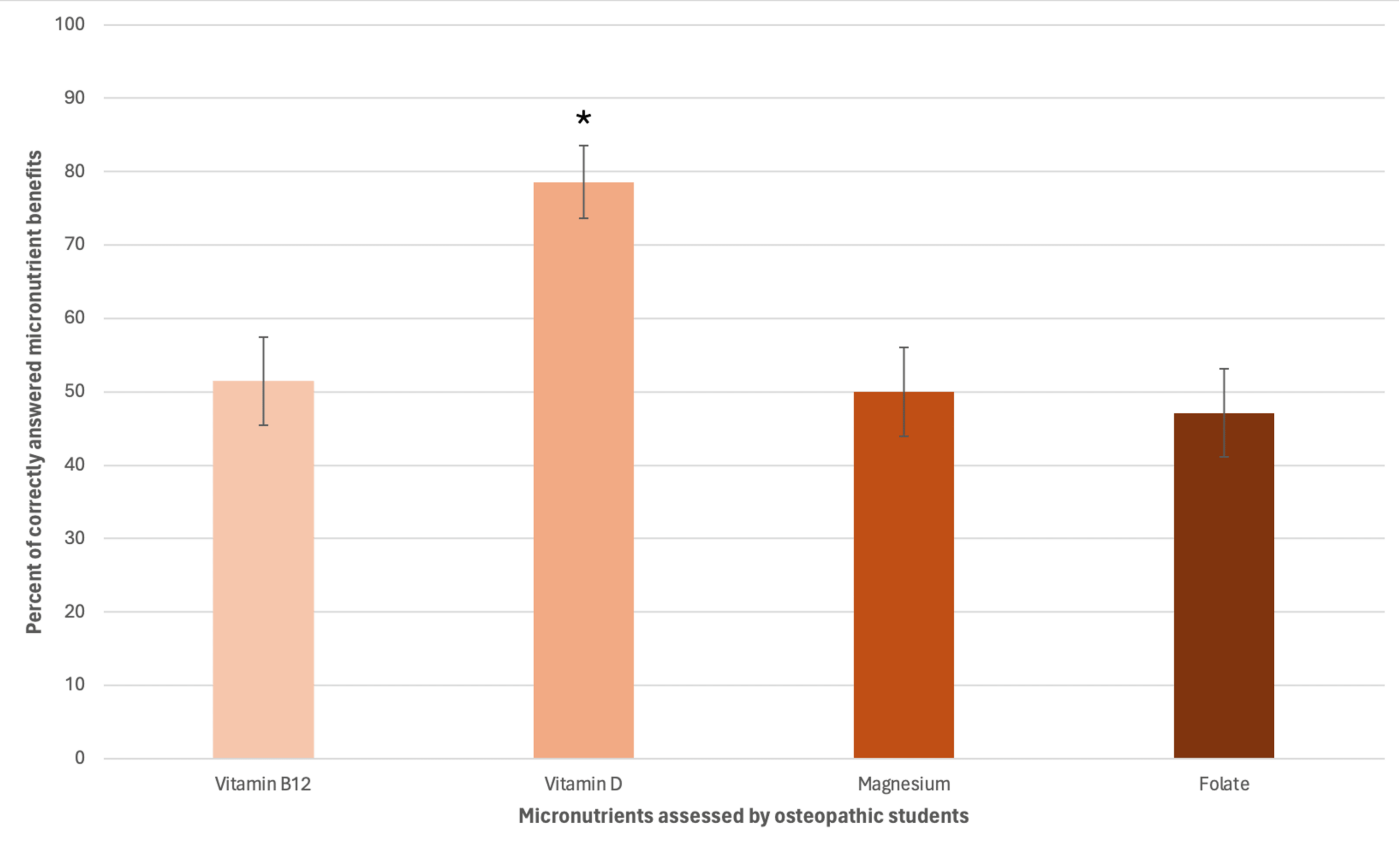
Micronutrient knowledge of all osteopathic students. ANOVA: analysis of variance, HSD: honestly significant difference.

An analysis of variance (ANOVA) was performed to compare the number of students (n = 70) who correctly answered questions regarding the specific benefits of vitamin B12, vitamin D, magnesium, and folate. The p-value for the ANOVA was <0.0001, indicating a statistically significant difference between the groups. A large effect size of 0.065 was calculated using eta-squared. A Tukey HSD test was performed to further analyze the differences identified in the ANOVA results. Significant differences were found between correct answers for vitamin D and those for vitamin B12, magnesium, and folate, with p-values of <0.01 for all. These results are displayed graphically in Figure 2.

Likelihood of future micronutrient recommendations

A four-point rating scale was administered to all students to assess the likelihood that they would recommend vitamin B12, vitamin D, magnesium, or folate to their future patients. The results of the rating scale are displayed in Table 3. The overall percentages of students who would recommend (sum of students who responded “somewhat likely” and “very likely”) vitamin B12, vitamin D, magnesium, and folate were 64 (91.4%), 67 (95.7%), 63 (90.0%), and 65 (92.9%), respectively.

**Table 3:** Likelihood of future micronutrient recommendation by osteopathic medical students. The large majority of students would recommend each micronutrient, meaning they are somewhat likely or very likely to recommend these vitamins and minerals in the future.

Likelihood of Future Patient Recommendation	n (%)
Vitamin B12	
Very unlikely	0 (0.0%)
Somewhat unlikely	6 (8.6%)
Somewhat likely	39 (55.7%)
Very likely	25 (35.7%)
Total that would recommend:	64 (91.4%)
Vitamin D	
Very unlikely	1 (1.4%)
Somewhat unlikely	2 (2.9%)
Somewhat likely	24 (34.3%)
Very likely	43 (61.4%)
Total that would recommend:	67 (95.7%)
Magnesium	
Very unlikely	1 (1.4%)
Somewhat unlikely	6 (8.6%)
Somewhat likely	41 (58.6%)
Very likely	22 (31.4%)
Total that would recommend:	63 (90.0%)
Folate	
Very unlikely	0 (0.0%)
Somewhat unlikely	5 (7.1%)
Somewhat likely	39 (55.7%)
Very likely	26 (37.1%)
Total that would recommend:	65 (92.9%)

Eagerness to learn more about micronutrients 

Students were asked if they would like to learn more about micronutrients in their medical school curriculum. The responses were a resounding yes, with 68 (97.1%) students showing interest in more extensive micronutrient education.

## Discussion

Self-rated nutritional education and confidence

The results of the degree of nutritional education the students received prior to taking the survey indicate that the large majority of students have had prior exposure to nutrition and the concept of micronutrients. Their confidence in their knowledge of nutrition is less robust, with most students selecting somewhat unconfident or only somewhat confident in their knowledge. Only one student stated they were very confident in their knowledge. This shows that while students may have attended a lecture or even taken a class in nutrition, their learning has not persisted throughout their medical education. Prior research supports this finding, with similar studies also indicating that additional nutrition education within medical school and alternative methods of teaching with greater lasting impact are needed to boost confidence levels in these students [20,21].

Micronutrient knowledge of pre-clinical vs. post-clinical students

All p-values were significant when comparing the micronutrient knowledge of pre-clinical students (OMS-I and OMS-II) to post-clinical students (OMS-III and OMS-IV). This indicates that students are retaining some of the nutritional education that is currently taught in the curriculum as they progress through medical school. Previous research assessing nutrition education growth throughout medical school corroborates this finding, showing that student knowledge increases with training and that students are motivated to learn [22]. Nutritional education holds importance and has staying power with students, and students are eager to possess this knowledge as they head into their clinical years.

Specific micronutrient knowledge of all students

The ANOVA comparing the number of students who correctly answered the specific benefits of vitamin B12, vitamin D, magnesium, and folate was found to be significant. The Tukey HSD test specified that students retain the most information about vitamin D compared to the other micronutrients. This indicates that more emphasis is being placed on the benefits of vitamin D compared to other micronutrients, either due to curriculum favoritism or increased awareness among the general public through national campaigns highlighting vitamin D deficiency [23,24]. This comparison helps to identify where the medical school curriculum needs to be supplemented, demonstrating a need for more lasting education detailing the benefits of vitamin B12, magnesium, and folate. All four of these vitamins are commonly deficient in the population and can have devastating impacts if insufficient, as stated in the introduction, so it is important for future physicians to have the ability to recognize and treat all of these micronutrient deficiencies.

Likelihood of future micronutrient recommendations

Survey results indicate that the large majority of students are likely to recommend these micronutrients to their future patients. While it is encouraging that students are inclined to recommend these critical nutrients, it highlights a gap in learning that must be rectified. Prior research suggests that providers often feel unprepared to recommend specific nutritional goals to their patients, despite believing in their importance [25]. These findings demonstrate that students are supportive of the solution, but they also must be informed of the magnitude of the deficiencies in our population and be able to identify the signs of specific vitamin deficiencies once they enter clinical practice.

Call for education

In line with the findings demonstrated in prior research, each of the findings of this study demonstrates a clear need for further education in micronutrient deficiency [18,19]. Improvement in the quantity and quality of micronutrient education for medical students will allow future physicians to become informed about these deficiencies and have the opportunity to apply this knowledge in their practices. In particular, vitamin B12, magnesium, and folate education should be bolstered for the reasons discussed above. The method of material introduction may vary based on curriculum structure and instructor preference, but a generally accepted learning method for long-term knowledge persistence is the practice of spaced repetition [26,27]. Introduction and reintroduction of this material over spaced time intervals, such as a nutrition lecture during each systems course, will aid retention of this knowledge and contribute to its application in clinical practice.

Limitations

A limitation of this study is the relatively small sample size (n = 70) compared to the ACOM student body of 806 individuals. Due to this low response rate, the power of the t-tests averaged 0.81 (with values between 0.69 and 1.0), and the power of the ANOVA test was 0.41. While the power of the ANOVA study is lower than anticipated, the large effect sizes found for both the t-tests and ANOVA contribute to the validity of the statistical findings in this study and support the presented conclusions. Opportunities for further investigation with a larger sample size are encouraged, but the authors are confident that the proposed conclusions are strong and valid. Another limitation is the lack of a formal validation process for the questionnaire prior to distribution, and, as a result, the precision and generalizability of the findings may be limited. This study also utilized self-reported survey data and is susceptible to subjective response bias, which may impact the findings. Future studies should aim to minimize response bias through validated and objective measures. Finally, this study was conducted at a single site, which may limit the generalizability of the findings to other settings. Future multi-site studies with larger sample sizes are recommended to extend these findings.

## Conclusions

The findings of this study demonstrate a resounding need for further education on micronutrient deficiency in undergraduate medical curricula. Specifically, efforts to enhance long-term retention of micronutrient knowledge are needed to improve the persistence of evidence learned throughout medical school. Expansion of micronutrient education has the potential to directly influence the clinical reasoning of future physicians, prompting transformation in patient care and improvement in the health of the nation as a whole.

## Appendices

**Table 4:** Questionnaire given to osteopathic medical students to assess knowledge of micronutrients

Question	Choice 1	Choice 2	Choice 3	Choice 4	Choice 5
1. Which medical school class are you?	OMS-I	OMS-II	OMS-III	OMS-IV	-
2. What is your education level?	None	No formal education, but have done personal research in nutrition	Have attended a lecture on nutrition	Have taken a nutrition class	Have a formal degree in nutrition
3. How confident are you in your education on nutrition?	Very unconfident	Somewhat unconfident	Somewhat confident	Very confident	-
4. How important do you think micronutrients are to overall health?	Very unimportant	Somewhat unimportant	Somewhat important	Very important	-
5. Please select the specific benefit of Vitamin B12.	Improves symptoms of migraine, Alzheimer’s disease, stroke, hypertension, cardiovascular disease, and type 2 diabetes mellitus	Prevention of anemia, nervous degeneration, and psychiatric illness	Prevention of anemia, cardiovascular disease, cancer, and cognitive function	Reduces fracture risk, cancer mortality, and risk of developing multiple sclerosis	-
6. Please select the specific benefit of Vitamin D.	Improves symptoms of migraine, Alzheimer’s disease, stroke, hypertension, cardiovascular disease, and type 2 diabetes mellitus	Prevention of anemia, nervous degeneration, and psychiatric illness	Prevention of anemia, cardiovascular disease, cancer, and cognitive function	Reduces fracture risk, cancer mortality, and risk of developing multiple sclerosis	-
7. Please select the specific benefit of Magnesium.	Improves symptoms of migraine, Alzheimer’s disease, stroke, hypertension, cardiovascular disease, and type 2 diabetes mellitus	Prevention of anemia, nervous degeneration, and psychiatric illness	Prevention of anemia, cardiovascular disease, cancer, and cognitive function	Reduces fracture risk, cancer mortality, and risk of developing multiple sclerosis	-
8. Please select the specific benefit of Folate.	Improves symptoms of migraine, Alzheimer’s disease, stroke, hypertension, cardiovascular disease, and type 2 diabetes mellitus	Prevention of anemia, nervous degeneration, and psychiatric illness	Prevention of anemia, cardiovascular disease, cancer, and cognitive function	Reduces fracture risk, cancer mortality, and risk of developing multiple sclerosis	-
9. How likely are you to recommend Vitamin B12 to your future patients?	Very unlikely	Somewhat unlikely	Somewhat likely	Very likely	-
10. How likely are you to recommend Vitamin D to your future patients?	Very unlikely	Somewhat unlikely	Somewhat likely	Very likely	-
11. How likely are you to recommend Magnesium to your future patients?	Very unlikely	Somewhat unlikely	Somewhat likely	Very likely	-
12. How likely are you to recommend Folate to your future patients?	Very unlikely	Somewhat unlikely	Somewhat likely	Very likely	-
13. Would you like to learn more about micronutrients and their impact on health during medical school?	Yes	No	-	-	-
